# Predictive factors for delayed hyponatremia after transsphenoidal surgery in patients with Rathke’s cleft cysts

**DOI:** 10.3389/fonc.2022.943666

**Published:** 2022-09-13

**Authors:** Kunzhe Lin, Zhijie Pei, Yibin Zhang, Tianshun Feng, Shousen Wang

**Affiliations:** ^1^ Fuzong Clinical Medical College of Fujian Medical University, Fuzhou, China; ^2^ Department of Neurosurgery, 900Hospital of Joint Logistics Support Force, Fuzhou, China; ^3^ Department of Neurosurgery, The First Affiliated Hospital of Fujian Medical University, Fuzhou, China; ^4^ Department of Neurosurgery, Dongfang Affiliated Hospital of Xiamen University, School of Medicine, Xiamen University, Xiamen, China

**Keywords:** Rathke’s cleft cysts, transsphenoidal surgery, delayed hyponatremia, magnetic resonance imaging, diaphragma sellae

## Abstract

**Purpose:**

We aimed to assess factors influencing the occurrence of delayed hyponatremia after transsphenoidal surgery in patients with Rathke’s cleft cysts (RCCs).

**Methods:**

We retrospectively collected the clinical data of patients who underwent transsphenoidal surgery for RCCs from January 2014 to January 2022. Univariate and multivariate analyses were used to determine the factors influencing the occurrence of postoperative delayed hyponatremia.

**Results:**

Of the 78 microscopic transsphenoidal surgery recipients with RCCs, 15 experienced postoperative delayed hyponatremia. There were 35 men and 43 women, and mean age was 43.75 ± 14.95 years. The clinical manifestations of RCCs were headache (62 cases, 79.5%), visual dysfunction (35 cases, 44.9%), endocrine dysfunction symptoms (12 cases, 15.4%). After transsphenoidal surgery, 93.5% (58/62) had improvements in headache, and 97.1% (34/35) had improved or resolved compressive visual symptoms. Delayed hyponatremia occurred on average on day 6.46 and lasted on average for 4.40 days. Logistic regression analysis showed that the independent influencing factor of delayed hyponatremia after transsphenoidal surgery in patients with RCCs was postoperative diaphragma sellae height.

**Conclusion:**

Postoperative diaphragma sellae height was identified as an independent influencing factor for delayed hyponatremia after transsphenoidal surgery in patients with RCCs.

## Introduction

Rathke’s cleft cysts (RCCs) are the most common incidentally discovered sellar lesions, followed by pituitary adenomas (1, 2). They are believed to derive from remnants of Rathke’s pouch, a dorsal invagination of the stomodeal ectoderm, typically positioned between the adenohypophysis and the neurohypophysis (3, 4). RCCs are usually discovered incidentally, but in a small number of cases, the cyst’s growth compresses the surrounding structures, leading to headaches or visual impairment (5). For symptomatic RCCs, surgical resection can be performed *via* a transsphenoidal approach (6).

Since the incidence of pituitary adenoma is higher than that of RCCs, there are many reports about delayed hyponatremia after transsphenoidal surgery in pituitary adenomas. Studies have shown that the incidence of delayed hyponatremia after a transsphenoidal pituitary approach in adenoma surgery can reach up to 15% (7). Delayed postoperative hyponatremia was defined as serum sodium levels of <135 mmol/L on or after postoperative day 3 (8). Hyponatremia can lead to a wide spectrum of clinical symptoms, ranging from subtle to severe or even life-threatening (9). The predictors of delayed hyponatremia after transsphenoidal surgery for pituitary adenomas have been studied in different medical centers. However, to date, the predictors of delayed hyponatremia for RCCs have not been reported.

Based on this lack of information, this study retrospectively collected clinical data related to RCCs, observed the changes in the diaphragma sellae and the pituitary stalk before and after surgery using magnetic resonance imaging (MRI), and analyzed the influencing factors of delayed hyponatremia after transsphenoidal surgery in RCCs.

## Methods

### Study cohort

The clinical data of patients who underwent transsphenoidal surgery for RCCs in Fuzong Clinical Medical College of Fujian Medical University from January 2014 to January 2022 were retrospectively collected. The ethics committee of the hospital approved the study, and all participants provided informed consent. The inclusion criteria were: 1) patients who underwent transsphenoidal surgery for the first time; 2) pathologically confirmed RCCs; 3) surgery performed by the same physician; and 4) the availability of complete clinical data. The exclusion criteria were: 1) RCCs complicated by other pituitary lesions, such as pituitary adenomas; and 2) history of radiotherapy or surgery in the pituitary region. The surgical indications were patients with RCCs with clinical symptoms such as repeated headache, visual impairment, and symptoms related to pituitary dysfunction (including short stature, decreased sexual function, diabetes insipidus, and menstrual disorders).

### Data collection

**Figure 1:**
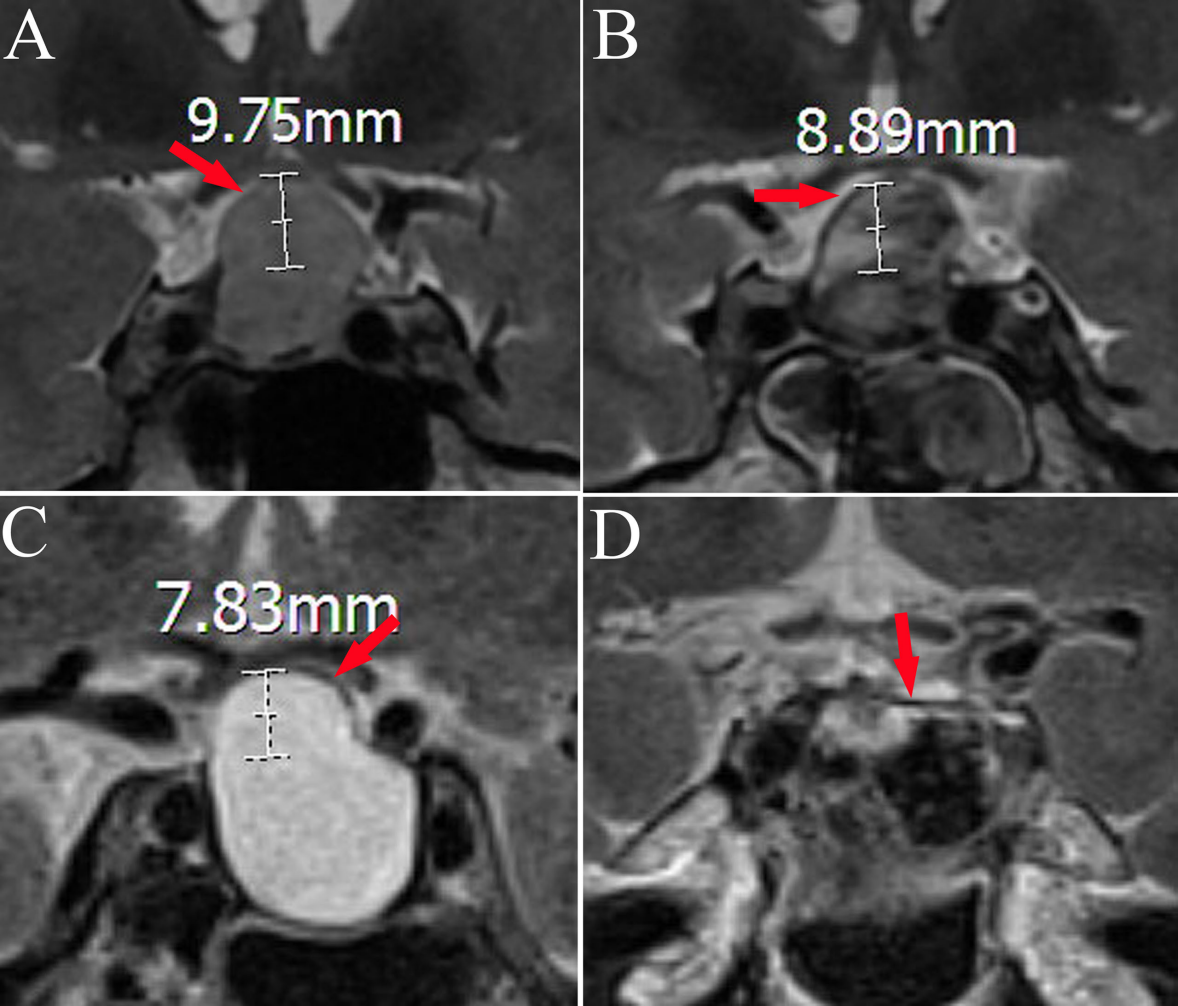
Magnetic resonance changes in the diaphragma sellae before and after transsphenoidal surgery in two cases. Panels **A, B** and panels **C, D** are from the same patient; diaphragma sellae (red arrow) **(A)**. Hypointense shadow of diaphragma sellae in preoperative T2-weighted imaging (diaphragma sellae height 9.75 mm). **(B)** The diaphragma sellae in postoperative T2-weighted imaging (diaphragma sellae height 8.89 mm). **(C)** Hypointense shadow of diaphragma sellae in preoperative T2-weighted imaging (diaphragma sellae height 7.83 mm). **(D)** The diaphragma sellae in postoperative T2-weighted imaging (diaphragma sellae height 0 mm).

**Figure 2:**
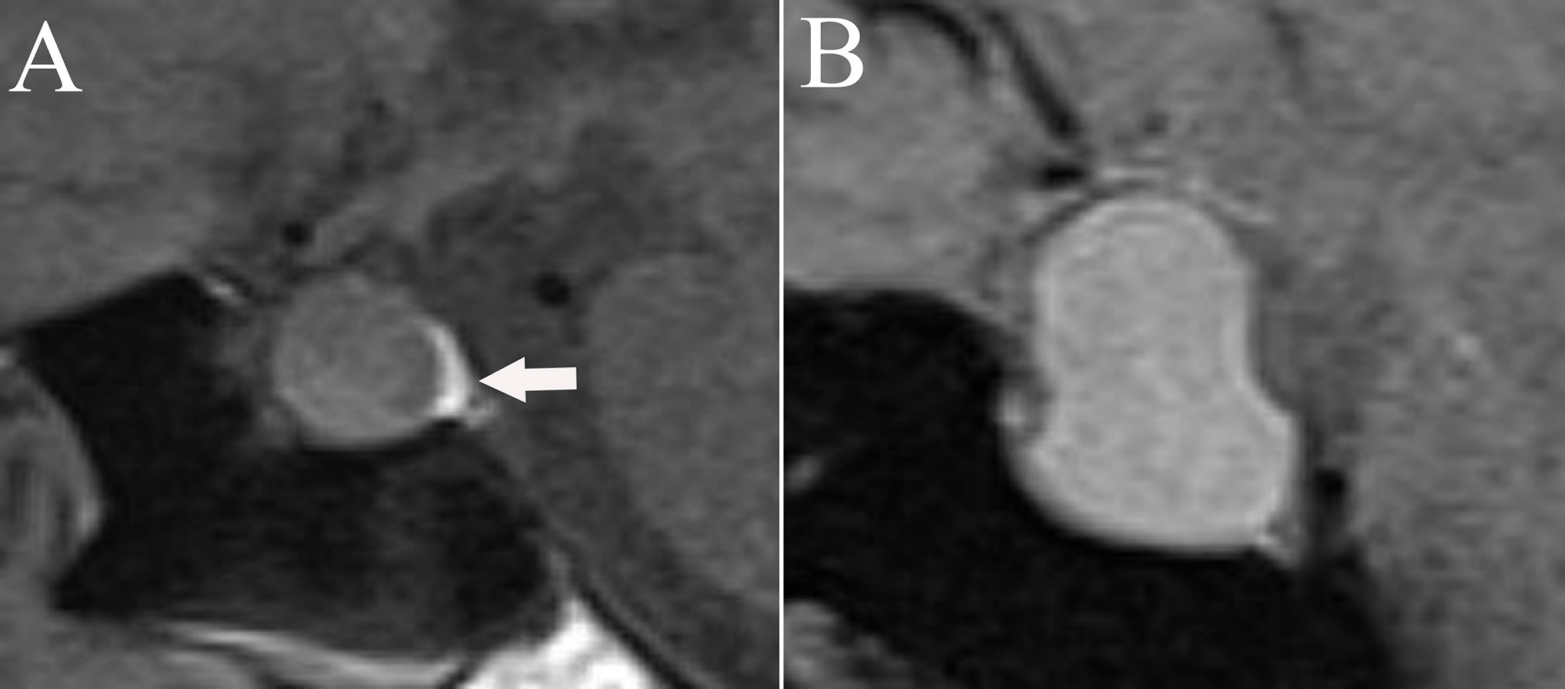
The appearance and location of posterior pituitary bright spots (white arrow) on T1-weighted images. **(A)** Posterior pituitary bright spots locates under the diaphragma sellae. **(B)** Posterior pituitary bright spots is nonvisable.

**Figure 3:**
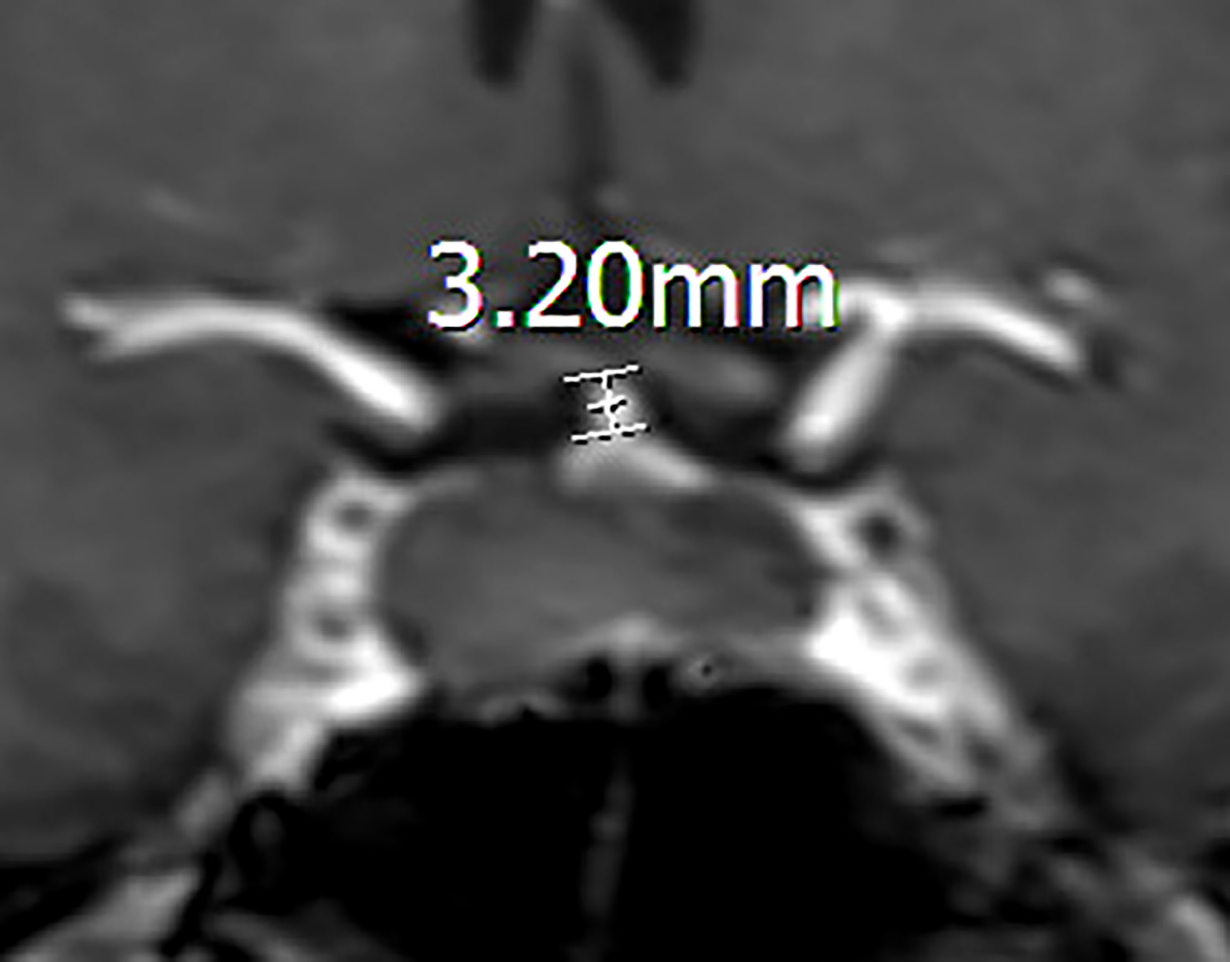
Coronal contrast-enhanced image showing the measurement of the pituitary stalk. The length of a preoperative pituitary stalk is 3.20mm.

We collected data on patients’ age, gender, MRI findings, and blood hormone levels (8:00 am, fasting) before and on the 1st postoperative day (including free triiodothyronine, free thyroxine, thyroid-stimulating hormone, cortisol, adrenocorticotropic hormone, prolactin (PRL), growth hormone, luteinizing hormone, and follicle-stimulating hormone). The pituitary region was scanned using 3.0 T MRI. Diaphragma sellae height was measured on T2-weighted images from the plane where the diaphragma sellae begins its elevation to the plane at which the highest point of the diaphragma sellae is located (**Figure 1**) (10). The appearance and location of posterior pituitary bright spots were observed on T1-weighted images (**Figure 2**) (11). The anteroposterior, transverse, and craniocaudal dimensions of RCCs and length of the pituitary stalk were measured on contrast-enhanced imaging (**Figure 3**). The volume of RCCs was calculated using the formula: anteroposterior dimension × transverse dimension × craniocaudal dimension/0.5 (12). MRI parameters were measured independently by a neurosurgeon and a neuroradiologist, and the average value was used for statistical analysis. Delayed hyponatremia was defined as blood sodium <135 mmol/L on or after the 3rd day post RCCs surgery.

### Perioperative management

A preoperative assessment of adenohypophyseal functioning was conducted, and the physiological supplementation of levothyroxine and glucocorticoid was administered to patients with low thyroid and adrenal axis functioning, respectively. All patients underwent cyst fenestration and drainage *via* the transsphenoidal approach under microscopy. Serum sodium levels were measured daily after surgery. All patients received sodium chloride solution (0.9%) or glucose injection (5%) perioperatively as maintenance fluid. Fasting was advised on the day of surgery. Maintenance fluid was infused at a rate of 100 mL/hour on postoperative day 0, reduced to 50 mL/hour on postoperative days 1–3, and usually stopped on postoperative day 4. Patients were allowed to start a liquid diet the day after surgery, gradually transitioning to a semi-solid diet. We routinely recorded the fluid intake and urine volume of each patient. Patients with diabetes insipidus (DI) were provided drinking water freely, and antidiuretic hormone analogs were used when necessary. For the diagnostic criteria of DI, refer to de Vries’ method (13). When the serum sodium was less than 135 mmol/L, fluid intake was restricted. According to blood sodium level, hyponatremia is divided into (9): mild (130–135 mmol/L), moderate (125–129 mmol/L), and severe (<125 mmol/L). For mild to moderate hyponatremia, liquid intake was limited to 1500–2000 mL per day; for severe hyponatremia, liquid intake was limited to 1000–1500 mL per day. We administered isotonic sodium chloride rehydration instead of hypertonic salt. Postoperative urine volume was monitored in clinically euvolemic patients with delayed hyponatremia in which adrenal insufficiency and hypothyroidism were excluded, and we presumed that inappropriate antidiuretic hormone secretion (SIADH) syndrome was the etiology (14). Patients who did not have surgical complications after transsphenoidal surgery for RCCs were routinely discharged on the 6th-7th day after surgery. Patients with complications, such as cerebrospinal fluid leakage, delayed hyponatremia, and DI, were discharged when they did not need further medical intervention.

### Statistical analysis

Statistical analyses were performed using IBM SPSS Statistics for Windows, ver. 19 (IBM Corp., Armonk, NY, USA). Quantitative variables are expressed as mean ± standard deviation or median (interquartile range). Continuous variables were compared using the standard Student’s t-test, and categorical variables were compared using Pearson’s chi-square test or Fisher’s exact test. Non-parametric tests were applied to variables with non-normal distributions. Univariate logistic regression was initially performed to identify variables of interest, and covariates with a P-value of <0.05 were incorporated into multivariable logistic regression models to identify independent risk factors for delayed hyponatremia. A P-value <0.05 was considered statistically significant.

## Results

### Patient characteristics

A total of 96 patients of RCCs underwent transsphenoidal surgery of which 78 met the inclusion criteria. Among them were 35 men and 43 women, with an average age of 43.75 ± 14.95 years. The clinical manifestations of RCCs were headache (62 cases, 79.5%), visual dysfunction (35 cases, 44.9%), endocrine dysfunction symptoms (such as irregular menses, libido changes, short stature, galactorrhea, polyuria, and polydipsia; 12 cases, 15.4%). Postoperative delayed hyponatremia occurred in 15 patients (the delayed hyponatremia group), including three cases of mild hyponatremia, five cases of moderate hyponatremia, and seven cases of severe hyponatremia. Delayed hyponatremia occurred, on an average, on day 6.46 and lasted an average of 4.40 days.

Of these 78 patients, cerebrospinal fluid leakage and DI occurred in 14 and 28 patients, respectively. Moreover, 93.5% (n = 58/62) had improved headache symptoms, and 97.1% (34/35) had improved or resolved compressive visual symptoms. All patients had resolved hyperprolactinemia, and most patients with preoperative endocrinopathies required ongoing medical replacement for hormonal deficiencies.

### Univariate analysis of risk factors for delayed postoperative hyponatremia

The comparison of different factors between the delayed hyponatremia group (n = 15) and the normonatremia group (n = 63) after transsphenoidal surgery in patients with RCC is shown in **Table 1**. Both groups had similar distributions regarding patient sex and age (P = 0.076 and 0.616, respectively). There was no significant between-group difference in the anteroposterior diameter, craniocaudal diameter, transverse dimension, or RCC volume. We also found that the postoperative diaphragma sellae height was much smaller in the delayed hyponatremia group than in the normonatremia group (P = 0.003), whereas there was no difference in preoperative diaphragma sellae height between the two groups (P = 0.074). Comparing the adenohypophyseal hormones of the two groups, we found that the pre- and postoperative PRL levels in the delayed hyponatremia group were significantly lower than those in the normonatremia group (P = 0.013 and 0.033, respectively).

**Table 1:** The comparison of factors between the delayed hyponatremia group and the normonatremia group.

Factors	Delayed hyponatremian=15	Normonatremian=63	*P* value
Age	43.00 (27.00, 56.00)	45.00 (36.00, 55.00)	0.616
Sex			0.076
Male	8	27	
Female	7	36	
Anteroposterior dimension, mm	10.91 (8.60, 12.13)	11.00 (9.67, 13.00)	0.462
Transverse dimension, mm	14.42 (10.98, 20.72)	12.90 (11.42, 15.85)	0.389
Craniocaudal dimension, mm	12.61 (10.30, 15.82)	13.19 (10.41, 17.30)	0.581
RCC volume, cm^3^	0.97 (0.66, 1.61)	0.94 (0.61, 1.48)	0.955
Preoperative DS height, mm	3.58 (0.00, 4.44)	4.44 (3.19, 6.80)	0.074
Postoperative DS height, mm	0.00 (0.00, 2.87)	3.83 (1.50, 6.08)	**0.003**
Intraoperative cerebrospinal fluid leaks			0.886
Yes	2	12	
No	13	51	
Postoperative DI			0.818
Yes	5	23	
No	10	40	
Preoperative serum sodium level, mEq/L	141.00 (137.00, 142.50)	139.00 (141.00, 143.00)	0.280
Preoperative prolactin, ng/ml	7.93 (3.81, 11.47)	11.17 (8.04, 18.38)	**0.013**
Postoperative prolactin, ng/ml	5.23 (4.11, 6.76)	6.94 (5.26, 10.14)	**0.033**
Preoperative FT3, pmol/L	4.73 (3.62, 5.32)	4.76 (4.16, 5.28)	0.634
Postoperative FT3, pmol/L	3.47 (2.94, 3.95)	3.52 (3.06, 3.91)	0.849
Preoperative FT4, pmol/L	14.80 (12.53, 16.48)	14.24 (13.09, 15.94)	0.980
Postoperative FT4, pmol/L	14.65 (13.07, 16.53)	15.54 (12.95, 16.83)	0.634
Preoperative TSH, mIU/L	1.09 (0.78, 1.34)	1.54 (1.02, 2.34)	0.066
Postoperative TSH, mIU/L	0.67 (0.36, 0.88)	0.57 (0.47, 0.93)	0.829
Preoperative cortisol, nmol/L	10.12 (3.00, 17.02)	14.49 (6.77, 18.33)	0.313
Postoperative cortisol, nmol/L	13.37 (10.45, 20.33)	21.54 (12.30, 32.55)	0.060
Preoperative ACTH, pg/ml	20.13 (9.02, 25.01)	22.70 (12.44, 36.02)	0.185
Postoperative ACTH, pg/ml	12.48 (7.13, 23.90)	18.21 (12.46, 33.51)	0.118
Preoperative GH, μg/L	0.52 (0.11, 2.61)	0.34 (0.11, 1.21)	0.319
Postoperative GH, μg/L	0.98 (0.68, 1.41)	0.60 (0.27, 1.76)	0.342
Preoperative LH, mU/ml	7.61 (1.71, 19.04)	5.73 (2.97, 11.56)	0.766
Postoperative LH, mU/ml	4.99 (1.86, 9.04)	4.27 (2.08, 7.66)	0.678
Preoperative FSH, mU/ml	4.73 (2.45, 22.65)	5.88 (4.26, 12.49)	0.708
Postoperative FSH, mU/ml	4.59 (3.01, 13.30)	5.36 (2.80, 10.39)	0.919

RCC, Rathke’s cleft cysts; DS, diaphragma sellae; DI, diabetes insipidus; FT3, Free triiodothyronine; FT4, free thyroxine; TSH, thyroid-stimulating hormone; ACTH, adrenocorticotropic hormone; GH, growth hormone. Values are median (interquartile range). Significant P values are shown in bold.

There were 27 cases of transient DI and one case of permanent DI. Most cases of DI occurred 1–2 days after surgery, with an average duration of 3.5 ± 1.7 days. In the normonatremia group, 23 patients had transient DI after surgery, four had serum sodium levels higher than 145 mmol/L, and no permanent DI occurred. In the delayed hyponatremia group, there were five cases of DI. Four of them presented with transient DI first, followed by hyponatremia. Another patient presented with transient DI, followed by 6 days of hyponatremia, which was then followed by permanent DI requiring long-term oral desmopressin. There was no difference in postoperative DI between the delayed hyponatremia and normonatremia groups (P = 0.818). In addition, there was no significant between-group difference in the incidence of intraoperative cerebrospinal fluid leakage (**Table 1**).

### Changes in the pituitary stalk

Among 78 RCC patients, the pituitary stalk was identifiable preoperatively in 50 cases and was unidentifiable in 28. The pituitary stalk was postoperatively identifiable in 54 cases and unidentifiable in 24. There was no difference in the average length of the preoperative pituitary stalk between the delayed hyponatremia and normonatremia groups (3.25 vs. 4.00 mm, P = 0.865). The average length of the postoperative pituitary stalk in the delayed hyponatremia group was greater than that in the normonatremia group (5.74 vs. 4.40 mm, P = 0.039; **Table 2**).

**Table 2:** Changes in the pituitary stalk after transsphenoidal surgery in patients with Rathke’s cleft cysts.

Factors	Delayed hyponatremian=15	Normonatremian=63	*P* value
Preoperative pituitary stalk			0.153
identifiable	12	38	
unidentifiable	3	25	
Postoperative pituitary stalk			0.052
identifiable	14	40	
unidentifiable	1	23	
Length of the preoperative pituitary stalk, mm	3.25 (2.99, 5.93)	4.00 (3.29, 4.73)	0.865
Length of the postoperative pituitary stalk, mm	5.74(4.40, 6.35)	4.40(3.80, 5.16)	**0.039**

Significant P values are shown in bold.

### Independent risk factors for delayed hyponatremia

We selected variables with P <0.05 in univariate analysis to be included in the logistic multivariate regression model. These variables were: postoperative diaphragma sellae height, preoperative PRL, and postoperative PRL. Logistic regression analysis showed that the independent influencing factor associated with delayed hyponatremia after transsphenoidal surgery in patients with RCCs was postoperative diaphragma sellae height (odds ratio = 0.692, 95% confidence interval = 0.525– 0.912; P = 0.009; **Table 3**).

**Table 3:** Multivariate logistic regression analysis of risk of delayed postoperative hyponatremia for Rathke’s cleft cysts.

Factors	OR	95% C I	*P* value
Postoperative DS height	0.692	0.525, 0.912	**0.009**
Preoperative prolactin Postoperative prolactin	0.925 0.900	0.782, 1.094 0.681, 1.191	0.362 0.462

OR, odds ratio; CI, confidence interval; DS, diaphragma sellae. Significant P values are shown in bold.

## Discussion

This study showed that the incidence of delayed hyponatremia after transsphenoidal surgery in patients with RCCs was 19.23%, similar to the incidence of delayed hyponatremia associated with pituitary adenomas. For the first time, we established the influencing factor of delayed hyponatremia after transsphenoidal surgery for RCCs.

The natural history of both symptomatic and asymptomatic RCCs is not clear (2). At present, the surgical indications for RCCs remain controversial. According to the size of the cyst, some scholars divided the condition into three categories (15): large symptomatic cysts warranting surgery, small incidental cysts requiring observation only, and intermediate size cysts, some of which may necessitate surgery and others that are likely asymptomatic and can be safely observed. Symptomatic RCCs manifests mainly as visual impairment, hypopituitarism, and headaches (16). Headaches are common symptoms in patients with RCCs. Among these, frontal, episodic, nonpulsating, bilateral headaches or deep retroorbital pain are the most common (2). Headaches does not correlate with the cyst size or location but is related to either high or isointense content on T1-weighted MRI, mucous cyst content, or intense chronic cyst wall inflammation (17). Headaches in patients with pituitary adenoma are caused by the traction and displacement of the diaphragma sellae by the suprasellar extension of the tumor (18).

Delayed hyponatremia after transsphenoidal surgery with a pituitary adenoma is a consequence of the mechanical disturbance of the pituitary stalk or posterior pituitary, resulting in the uncontrolled release of antidiuretic hormone (ADH) and the occurrence of SIADH (19). Similarly, the cause of postoperative delayed hyponatremia for RCCs can also be explained by SIADH. In this study, delayed hyponatremia occurred on postoperative days 6–7, similar to the time window for SIADH after transsphenoidal surgery for pituitary adenoma. Regarding craniopharyngioma, Liu et al. reported that the incidence of delayed hyponatremia after surgery was as high as 44.8% (20). The main causes of postoperative hyponatremia are SIADH and cerebral salt-wasting syndrome (21).

Age and gender are predictors of delayed hyponatremia after transsphenoidal surgery for pituitary adenoma (22–25). However, we showed that age and gender had no predictive value for the occurrence of delayed hyponatremia in RCCs. We found that the greater the height of the diaphragma sellae after surgery, the lower the risk of postoperative delayed hyponatremia. In most pituitary adenomas, the upper surface of the tumor is covered with an intact diaphragma sellae, which subsides after tumor resection *via* a transsphenoidal approach (26). Likewise, RCCs is similar to this. During the process of cyst enlargement, the diaphragma sellae is pushed upward, and the subsidence of the diaphragma sellae occurs after the cyst contents are removed. The diaphragma sellae is continuous anteriorly with the dura mater covering the anterior cranial fossa and posteriorly with the dura covering the dorsum sellae and clivus (27). To a certain extent, the lower postoperative elevation of the diaphragma sellae reflects its obvious subsidence and a higher risk of delayed hyponatremia. The reason may be that the subsidence of the diaphragma sellae in these patients is obvious, pulling and causing damage to the pituitary stalk and leading to excessive ADH secretion, as shown in previous studies (10). We found that, postoperatively, the average length of the pituitary stalk was significantly greater in the delayed hyponatremia group than in the normonatremia group.

Previous studies have shown that hypofunction of the thyroid or adrenal axes is one of the causes of hyponatremia (28, 29). In our study, we performed adequate hormone supplementation for patients with hypopituitarism. Therefore, there was no difference in the levels of hormones related to the thyroid and adrenal axes between the normonatremia and hyponatremia groups. The PRL reference range is 5–25 ng/mL in women and 5–18 ng/mL in men (30). We found that the postoperative PRL level in the hyponatremia group (5.23 ng/mL) was lower than that in the normonatremia group (6.94 ng/mL), but its average value fell within the normal reference value range.

In our center, we performed preoperative MRI evaluations on all patients with RCCs. MRI is helpful in differentiating RCCs from pituitary adenomas, and different sequences of MRI can be used to better observe the cystic fluid. In the postoperative follow-up of RCCs, attention should be paid to observe whether hormone levels have recovered, whether visual acuity has improved, and whether there is recurrence, through MRI reexamination.

This study has some limitations. As the incidence of RCCs is lower than that of pituitary adenomas, retrospective studies have resulted in a relatively limited sample size. In addition, all cases in our center underwent surgery under microscopy. The effect of different surgical tools (endoscope vs. microscope) on the occurrence of delayed hyponatremia after transsphenoidal surgery with RCCs requires further multi-center research.

## Conclusions

This study is the first to predict the occurrence of delayed hyponatremia after transsphenoidal surgery in patients with RCCs; the smaller the postoperative elevation of the diaphragma sellae, the higher the risk of delayed hyponatremia.

## Data availability statement

The original contributions presented in the study are included in the article/Supplementary Material. Further inquiries can be directed to the corresponding author.

## Ethics statement

The studies involving human participants were reviewed and approved by Fuzong Clinical Medical College of Fujian Medical University. The patients/participants provided their written informed consent to participate in this study.

## Author contributions

KL: Data curation, Writing original draft. ZP: Data curation, Writing original draft. YZ: Data retrieval, statistics. TF: Data retrieval, statistics. SW: designed the study and revised the manuscript. All authors reviewed the manuscript.

## Funding

This work was supported by Innovation Joint Fund Project of Fujian Province, China (No. 2019Y9045).

## Conflict of interest

The authors declare that the research was conducted in the absence of any commercial or financial relationships that could be construed as a potential conflict of interest.

## Publisher’s note

All claims expressed in this article are solely those of the authors and do not necessarily represent those of their affiliated organizations, or those of the publisher, the editors and the reviewers. Any product that may be evaluated in this article, or claim that may be made by its manufacturer, is not guaranteed or endorsed by the publisher.
